# Morphological and conceptual influences on the real-time comprehension of optional plural marked sentences in Yucatec Maya

**DOI:** 10.3389/fpsyg.2023.1135474

**Published:** 2023-08-23

**Authors:** Lindsay K. Butler

**Affiliations:** Department of Speech, Language and Hearing Sciences, University of Connecticut, Storrs, CT, United States

**Keywords:** morphology, plural, sentence comprehension, Yucatec Maya, psycholinguistics

## Abstract

**Introduction:**

Psycholinguistic research often focuses on Indo-European and other commonly studied major languages, while typologically diverse languages remain understudied. In this paper, we examine the morphological and conceptual influences on the real-time comprehension of optional plural-marked sentences in Yucatec Maya, an indigenous language of Mexico with a less commonly studied optional plural marking system.

**Methods:**

Fifty-one speakers of Yucatec Maya participated in a picture-sentence matching experiment carried out in the Yucatan Peninsula of Mexico. Pictures of one, two, or seven humans or animals depicting an intransitive action (conceptual number) were paired with auditorily presented sentences that had no plural marking, one plural, or two plurals (morphological number). Participants indicated by key press whether the picture and the sentence were an acceptable match, and decision time was recorded.

**Results:**

In the analysis of decision (yes versus no) and accuracy, morphological and conceptual factors interacted. In the analysis of decision time, however, morphological plural marking, but not conceptual number, led to faster decisions.

**Discussion:**

In light of previous work on the role of conceptual factors in the computation of number agreement, the interaction between conceptual and morphological factors suggests that a language with optional plural marking (or low “morphological richness”) is associated with high conceptual influence on sentence comprehension. Importantly, the results of this study expand the empirical base of language types that have been investigated using psycholinguistic methods.

## 1. Introduction

A few decades of research into the factors that drive erroneous or mis-matched number agreement have spanned the disciplines of psycholinguistics (e.g., Bock and Miller, 1991; Vigliocco et al., 1996b; Bock et al., 2001; Eberhard et al., 2005, *inter alia*) and linguistics (e.g., Jespersen, 1924; Kimball and Aissen, 1971; Quirk et al., 1985; Francis, 1986; den Dikken, 2001, *inter alia*). Called the “agreement attraction effect”, e.g., “The key to the cabinets are on the table”, this phenomenon has been central to language production research showing how conceptual information (e.g., numerosity) may drive the agreement attraction effect as, conceptually, multiple cabinets are more likely to have multiple keys rather than one key that opens all cabinets (Bock and Miller, 1991). Several early studies replicated this effect (Bock and Eberhard, 1993; Franck et al., 2002; Bock et al., 2004; Eberhard et al., 2005). The agreement attraction effect has drawn attention in language comprehension research (e.g., Wagers et al., 2009; Kreiner et al., 2013; Tanner et al., 2014; Lago et al., 2015, *inter alia*) because of its central role in grammatical encoding and retrieval. Due to the ubiquity of agreement and agreement attraction, it has been investigated in several languages other than English, though it has rarely been investigated in non-Indo-European languages.

With an increase in cross-linguistic research, the question of whether number agreement processing is affected by the same types of information across different languages has come into question (Bock et al., 2001, 2006; Lorimor et al., 2008; Foote and Bock, 2012). In particular, conceptual information seems to have different effects across diverse language types. For example, languages like Spanish and French show higher percentages of plural attraction in response to more conceptually plural antecedent noun phrases, like *la etiqueta en las botellas*, “the label on the bottles” in Spanish, compared to English (Vigliocco et al., 1996a,b). But, there is evidence that in Russian and German, conceptual effects on number agreement processing, as in *bilet na kontserty* “the ticket to the concerts”, do not elicit as many plural continuations as in English or Spanish due to language-specific grammatical factors (Berg, 1998; Lorimor et al., 2008).

One prominent approach to explaining this cross-linguistic variation is the morphological richness hypothesis which proposes a link between the richness of the morphological paradigms of a language and the extent to which conceptual information may intrude on agreement processing. There are, however, contrasting findings in studies on morphological richness. Vigliocco and Hartsuiker (2002) and Foote and Bock (2012) refer to these two divergent hypotheses as the “maximalist” and “minimalist” approaches. The maximalist hypothesis predicts that languages with richer inflectional systems will show an increased influence of conceptual number due to the salient role of meaning in the computation of agreement morphology (Vigliocco et al., 1996b). On the other hand, the “minimalist” hypothesis predicts that languages with richer inflectional morphology will show a decreased influence of conceptual number due to the salience of grammatical specifications of inflectional morphemes which cancel the effects of number meaning (Eberhard et al., 2005).

The goal of the present study is to examine the effects of conceptual and morphological number information on sentence comprehension in an understudied language type (with optional plural marking). Plural marking and number agreement are optional in Yucatec Maya (Butler, 2012). These features mean that plural marking in Yucatec Maya is not rich. We aim to use this property to directly test the influence of within-language morphological richness (contrasting no plural, one plural, two plurals) on the extent to which conceptual number information influences the comprehension of sentences with plural referents. One specific domain of conceptual number that has been shown to influence number agreement processing is numerosity, the quantity of units or individuals (Bock and Eberhard, 1993; Bock et al., 2012). We use numerosity of a nominal reference in an intransitive sentence (e.g., “The boy is writing”) to examine how conceptual number information influences the processing of optional number marking in Yucatec Maya. There is an important rationale for using numerosity in intransitive sentences in Yucatec Maya. In a complex noun phrase with the first noun singular and the second noun plural (as in “The key to the cabinets IS/ARE on the table”), the plural on the second noun is ambiguous as to which noun it modifies. It can mean either “The key to the cabinets” or “The keys to the cabinet”, so this paradigm used in previous psycholinguistic studies with speakers of Indo-European languages cannot be replicated with speakers of Yucatec Maya.

The primary goal of the current study is to elucidate the relationship between morphological and conceptual number information in sentence comprehension in a less-commonly studied, optional plural marking language, Yucatec Maya. The secondary goal of this paper is to address the implications of the effects of conceptual and morphological number in an optional plural language for the morphological richness hypothesis. Before outlining the methods, we provide background discussion of the linguistic properties of Yucatec Maya relevant to optional plural marking within sentences, since Yucatec Maya is a less-commonly studied language.

## 2. Background

### 2.1. The linguistic typology of number marking

Number is a common linguistic category across the languages of the world as discussed in in-depth typological studies (Mithun, 1999; Corbett, 2000). Most better-known languages, such as English and Spanish, have obligatory nominal plural marking, so plural morphology must be used every time a speaker mentions a nominal referent that refers to a plurality (more than one). Similarly, in these types of languages, number agreement between the subject and verb is obligatory. Every time a speaker uses a plural noun, he or she must mark agreement for number on the verb. Some languages, however, lack a nominal plural marker and subject-verb agreement for number. Other languages have a nominal plural marker, but its use is optional. In yet other languages the use of the nominal plural is conditioned by the animacy of the noun. For example, only human nouns are marked with the plural, and human nouns are obligatorily marked for plurality. In other languages, like Yucatec Maya, the nominal plural may be used with human and other animate nouns (Butler, 2012), and its use is optional.

**Figure 1:**
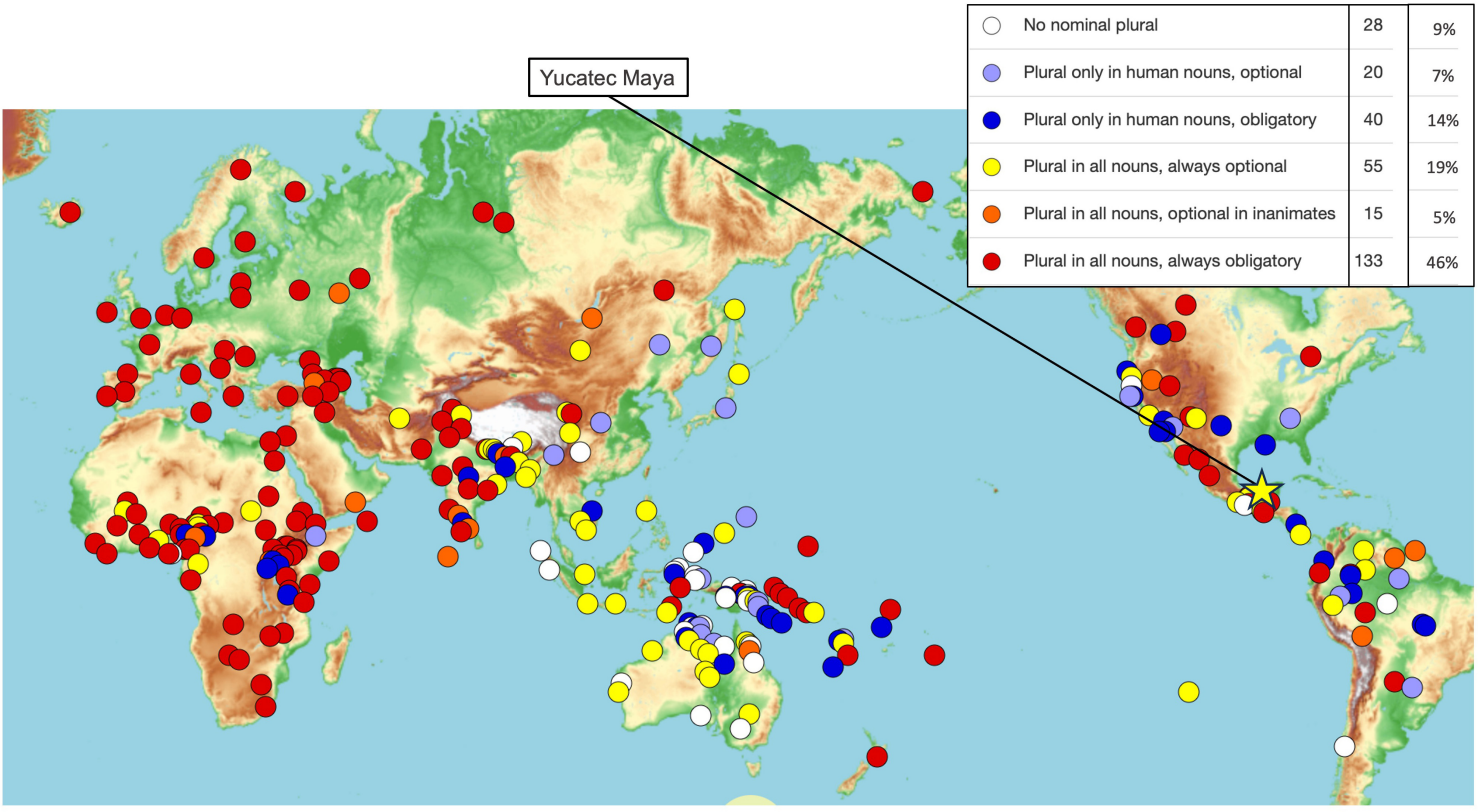
The typology of nominal plural marking from the World Atlas of Language Structures online (Haspelmath, 2013), reproduced in accordance with a Creative Commons Attribution 4.0 International License, yellow star added to highlight Yucatec Maya.

A query of the World Atlas of Language Structures (Haspelmath, 2013) resulted in six different language types in the domain of nominal plural marking. Obligatory plural marking that occurs in more commonly studied languages such as English and closely related Indo-European languages constituted 46% of the 291 languages surveyed. Languages that have no nominal plural morphology made up 9% of the languages surveyed. Languages that have optional plural marking for all nouns constituted 19% of the languages surveyed. The other language types had obligatory or optional plural marking only for certain classes of nouns (optional, only on human nouns: 7%; obligatory, only human nouns: 14%; optional, only in inanimates: 5%) (Haspelmath, 2013) (shown in Figure 1).

Though obligatory number agreement languages make up the most common number marking type, they do not represent the majority. Five other language types make up 54% of number marking language types. Much of this diversity has not been examined in well-controlled psycholinguistic studies due to a variety of challenges inherent in cross-linguistic psycholinguistics (Christianson and Ferreira, 2005; Norcliffe et al., 2015a). Despite challenges, languages with different number marking systems may be able to uniquely address questions of number agreement processing, such as the role of morphological richness. More generally, examining different language types will expand the empirical foundation of models of human language processing.

Psycholinguists have long been interested in what information is available at different processing stages and whether this can vary across different languages. Agreement processing provides a window into what information is available during positional processing, the stage at which inflectional morphology is hypothesized to be processed. The current study takes advantage of the optionality of plural marking in Yucatec Maya to examine cross-linguistic differences in the flow of information during sentence production and comprehension, a conclusion that is supported by research on agreement in the sentence (Foote, 2006; Lorimor et al., 2008) and noun phrase domains (Costa, 1999; Schiller and Caramazza, 2003). In the next section, we briefly outline the major linguistic properties of optional number marking in Yucatec Maya.

### 2.2. Number marking in Yucatec Maya

In Yucatec Maya, the use of the plural morpheme is optional—a sentence with no plural marking, such as (1a.) below, may be interpreted as referring to a singular entity (the dog) or to a plurality (the dogs). When a speaker of Yucatec Maya uses the plural, as in (1b.) through (1d.), it refers unambiguously to a plurality (see Butler, 2012 for more characteristics of optional of plural marking in Yucatec Maya).^1^

1. a. Le péek-o' táan u toj-oldet dog-d2 prog a3 bark-inc “The dog is barking.” or “The dogs are barking.”
b. Le péek-o'ob-o' táan u toj-ol det dog-pl-d2 prog a3 bark–inc “The dogs are barking.” not: “The dog is barking.”
c. Le péek-o' táan u toj-ol-o'ob det dog-d2 prog a3 bark-inc-pl “The dogs are barking.”not: “The dog is barking.”
d. Le péek-o'ob-o' táan u toj-ol-o'ob det dog-pl-d2 prog a3 bark-inc-pl “The dogs are barking.” not: “The dog is barking.”

In Yucatec Maya, plural marking is optional, and if a plural morpheme is used on the noun, it does not need to be used on the verb. Native speaker judgments (Butler, 2012) and sentence production experiments (Butler and Couoh Pool, 2018) have shown that plural marking mis-matches in Yucatec Maya are judged to be grammatical and are observed in sentence production tasks. The sentence in (1b.) shows that a speaker may produce a plural on the noun without producing an agreeing, co-varying plural morpheme on the verb of an intransitive sentence. Similarly, the sentence in (1c.) shows that a speaker of Yucatec Maya may produce the plural on the verb without producing an agreeing, co-varying plural on the noun. While early work suggested that animacy drove the use of the plural morpheme (Lucy, 1992), psycholinguistic studies have found no effect of animacy on the use of plural with human compared to animal reference (Butler, 2012). The production of the plural to is know to be influenced by numerosity and higher levels of education in Spanish (an obligatory plural language) (Butler and Couoh Pool, 2018).

A few additional linguistic properties of Yucatec Maya are noteworthy because they complicate the use of traditional psycholinguistic methods of investigating the processing of number marking. Yucatec Maya is a language that lacks a copular (“to be”) verb, so the study of optional number co-variation on the noun and verb in this language must be based on non-copular verbs. Most studies on the agreement attraction effect, however, present sentences with a copular verb involving one or more prepositional phrases and/or subordinate clauses (e.g., “The key to the cabinets *are* on the table” (Bock and Miller, 1991). In addition, stimulus sentences from traditional number agreement processing studies may not translate well into typologically diverse languages like Yucatec Maya. These stimuli often rely heavily on culturally-specific vocabulary for professions, such as pianist or essayist, that may not have natural translations into other languages. Since frequently occurring words are processed more quickly and may affect lexical selection in sentence production (e.g., Oldfield and Wingfield, 1965; Caramazza and Hillis, 1990; Dell, 1990) and comprehension (e.g., Ferreira et al., 1996; MacDonald, 1997), poorly translatable stimuli could present a major confound for psycholinguistic studies with speakers of Yucatec Maya. For these reasons, we focus on numerosity as a measure of conceptual effects on the processing of optional plural morphology in simple, intransitive sentences with an event-denoting verb.

The goal of the current study is to elucidate the relationship between morphological and conceptual number information in sentence comprehension in a less-commonly studied, optional plural marking language (Yucatec Maya). This paper also aims to address the implications of the effects of conceptual and morphological number in an optional plural language for the morphological richness hypothesis. The specific questions that this study aims to address are:

1. How does morphological information (the absence or presence of an optional plural morpheme) affect real-time sentence comprehension in speakers of Yucatec Maya?
2. How does conceptual information (pictures of one, two or seven items) affect real-time sentence comprehension in speakers of Yucatec Maya?
3. Do morphological and conceptual information interact in real-time sentence comprehension in speakers of Yucatec Maya?

Since Yucatec Maya does not have a rich inflectional system for number morphology, the maximalist hypothesis would predict little or no influence of conceptual information on real-time sentence comprehension. On the other hand, the minimalist hypothesis would predict that the less rich morphological number system of Yucatec Maya would lead to a greater influence of conceptual number in real-time sentence processing. If however, morphological and conceptual information interact in real-time sentence comprehension among speakers if Yucatec Maya, this may suggest a more nuanced approach to the relationship between form and meaning that may become more evident as psycholinguistic research expands its empirical base to typologically diverse languages.

## 3. Methods

### 3.1. Participants

Fifty-one speakers of Yucatec Maya from the community of Popolá, Yucatán, México (30 females and 21 males) between the ages of 10 and 65 (*M* = 22.8, *SD* = 12.2) participated in the experiment. Participants ranged in level of education from no formal education to some college (with a mean of an eighth grade level of education (*M* = 8.0, *SD* = 4.0). While participant age range was large, younger and older participants were well matched on level of education (as older participants tended to have lower levels of education). Education, rather than age, has been shown to be a stronger predictor of the use of optional plural marking in sentence production among speakers of Yucatec Maya (Butler and Couoh Pool, 2018). Nonetheless, the large age range should be taken into consideration as data from children and adults are analyzed together. Participants were paid 50 Mexican pesos for their participation, which lasted no longer than 30 min. All participants provided verbal assent in Yucatec Maya with the author and a speaker of Yucatec Maya and member of the community. The data were collected in 2016 in accordance with the Declaration of Helsinki and approved by the Institutional Review Board at Pennsylvania State University when the author was affiliated with that institution.

### 3.2. Procedure

The experiment was carried out on a MacBook Pro with a 15-inch screen diagonal using the PsychoPy software (Peirce, 2009) in an unoccupied room in the home of a Yucatec Maya-speaking consultant. Participants were seated in a chair at a small table. The table held the laptop and attached speakers. The auditory stimuli were delivered over speakers (rather than through the use of headphones) since the use of headphones or headsets was judged to be culturally inappropriate by the consultants. The doors of the room were closed while the participant was carrying out the experiment, and a Yucatec Maya-speaking consultant remained in the room or just outside the door. The participant was given instructions in Yucatec Maya. Participants were instructed to look at the picture and listen to the sentence played at the same time. The participant was told that he or she would be asked to decide if the picture and the sentence were a good match (with a response of “yes” or “no”). Using the PsychoPy software, the start time of the sentence presentation and the time of the button press were recorded. The participant was instructed to use his or her pointer finger to press a key to indicate his or her decision. A large blue sticker was placed on the bottom right corner of the laptop. The participant was instructed to keep the pointer finger on the large blue sticker at all times except when responding. The left arrow key was covered with a red sticker, and the right arrow key was covered with a green sticker. The participant was then told that the red/left key was to be pressed for a response of “no,” and the right/green key was to be pressed for a response of “yes.” The consultant watched the participant complete four training trials. After the four training trials, there was a break so that the consultant could clarify the instructions and/or ask if the participant had any questions.

### 3.3. Materials

The experimental stimuli included pictures of one, two and seven humans or animals depicting an intransitive action (see Table 1). All noun referents were countable nouns and not mass nouns. These pictures were combined with three sentence conditions [no plural, one plural (varying plural marking on the noun or verb), and plural on both the noun and verb] (see Table 2). The picture stimuli were combined with the three sentence conditions and pseudo-randomized in a Latin Square design into three experimental lists each consisting of 24 items and 36 fillers. The stimulus sentences were read and recorded by a linguist and native speaker of Yucatec Maya with an event but natural intonation. The stimulus sentences were presented auditorily at the same time as the stimulus pictures.

**Table 1:** Example picture stimuli by numerosity and humanness.

**Numerosity**	**One**	**Two**	**Seven**
Human	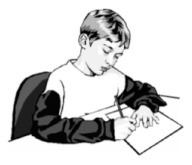	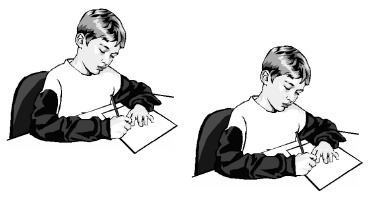	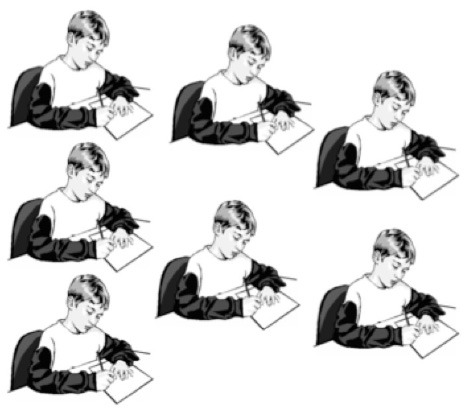
Animal	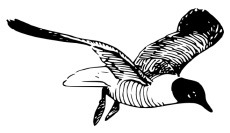	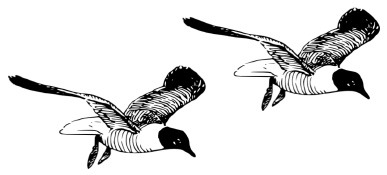	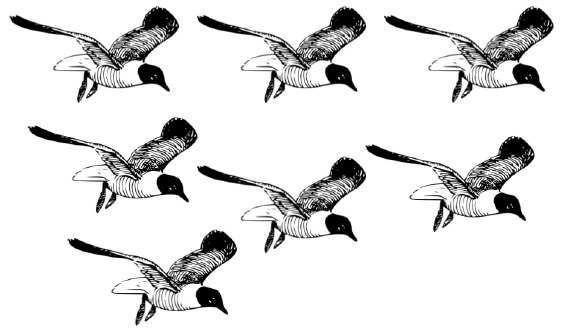

**Table 2:** Example sentence stimuli by plural marking condition.

**Sentence plural condition**	**Example**	
No plural	Le xibpal-o' táan u tsiib.	
	def boy-d2 prog a3 write	
	“The boy is writing.” / “The boys are writing.”	
One plural	Le xibpal-**o'ob**-o' táan u tsiib.	Le xibpal-o' táan u tsiib-**o'ob**.
	def boy-**pl**-d2 prog a3 write	def boy-d2 prog a3 write-**pl**
	“The boys are writing.”	“The boys are writing.”
Two plurals	Le xibpal-**o'ob**-o' táan u tsiib-**o'ob**.	
	def boy-**pl**-d2 prog a3 write-**pl**	
	“The boys are writing.”	

The experiment began with 16 practice trials. A high number of practice trials were included to stabilize decision times for the target items by allowing participants plenty of practice. Many of the participants have little experience with psycholinguistic experiments in contrast to a typical population of university students in Western countries in which Indo-European languages are spoken (cf. Henrich et al., 2010). In addition, some of the participants had little or no experience using a computer. During the practice trials, the participant was allowed to ask questions. Additionally, the participants were allowed to re-start the practice trials in order to clarify any questions or doubts about the instructions.

### 3.4. Exclusions

One participant's data was removed from the analysis for having an accuracy rate of under 50% and suspected cognitive and/or language impairment as judged by two Yucatec Maya speaking members of the community who were assisting with the research. One item was removed from the analyses due to experimenter error. Excluding one participant and one item resulted in a loss of 47 responses (3.8% of the total responses).

## 4. Planned analyses

For the analysis of decision time, we subtracted total response time from time of the stimulus sentence (since participants were presented with the auditory sentence and picture simultaneously). Three outcomes were analyzed in three separate models: (1) acceptability decision, (2) decision accuracy and (3) decision time. The predictor variables for all outcomes were: (1) conceptual number and (2) morphological number. The variable conceptual number was Helmert coded to investigate two contrasts: (1) the mean of one item pictured (no numerosity) vs. two items pictured (low numerosity) and (2) the mean of two items pictured (low numerosity) vs. seven items pictured (high numerosity). Helmert coding allowed us to test one vs. two items (singular vs. low numerosity) and two vs. seven items (low vs. high numerosity), rather than giving equal weight to one vs. two vs. seven, which would not have the same effect of testing the graded effects of numerosity in the statistical model. Along the same lines, the variable morphological number was contrast coded as such: (1) the mean of no plural marking (no morphological number information) vs. one plural (low morphological number information) and (2) the mean of one plural (low morphological number information) vs. two plurals (high morphological number information). Contrast coding was applied in this way to test for interactions between all levels of the predictor variables without resorting to *post hoc* tests. The analyses were carried out using R (R Core Team, 2022) and the *lme4* package (Bates et al., 2015) to construct mixed effects regression models (Breslow and Clayton, 1993; Baayen et al., 2008) of the relationship between numerosity and plural marking in predicting acceptability decisions, accuracy and decision time. As Spanish is widely spoken in the Yucatan Peninsula, we assume that participants varied in their proficiency in Spanish. To account for potential individual influences such as varying use of a second language, we used mixed-effects models with random intercepts for subjects and items. The formulas for computing these models in R were:

1. Model 1 <- lme(acceptability decision ~ Conceptual Number * Morphological Number + (1|item) + (1|subject), data = d, REML = FALSE)
2. Model 2 <- lme(accuracy ~ Conceptual Number * Morphological Number + (1|item) + (1|subject), data = d, REML = FALSE)
3. Model 3 <- lme(decision time ~ Conceptual Number * Morphological Number + (1|item) + (1|subject), data = d, REML = FALSE).

We first constructed linear models to verify that the participant's age, level of educational attainment and the order of presentation of stimuli did not significantly predict acceptability decision, accuracy, or decision time. None of these variables reached significance. We then conducted the three planned analyses and ran full models with random intercepts for subjects and items (Barr et al., 2013). We report the maximally converging models (Barr et al., 2013) that did not show evidence of overparameterization (fixed effect *r*s < 0.65). Models were compared in a likelihood ratio test with the argument “reml = false” (Pinheiro and Bates, 2000; Bolker et al., 2009; Winter, 2013).

## 5. Results

### 5.1. Acceptability decision

**Figure 2:**
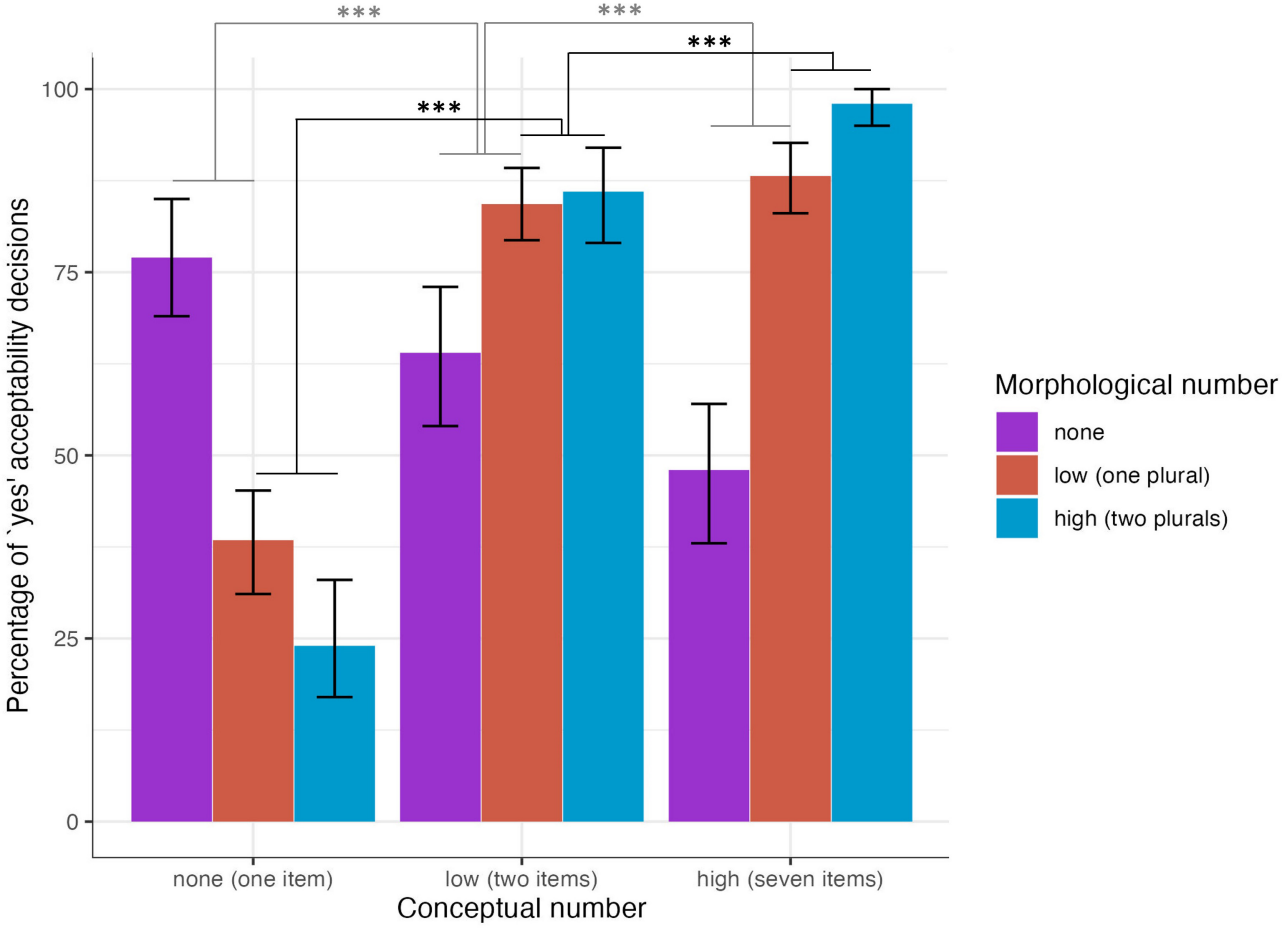
Percentage of “yes” acceptability decisions by morphological condition for the two and seven conceptual conditions (error bars indicate bootstrapped 95% confidence intervals). *p < 0.05, **p < 0.01, ***p < 0.001.

We analyzed to what extent plural morphology and numerosity predicted a participants' decisions (“yes” vs. “no”) that the auditorily presented sentence matched the picture presented. The results of the mixed effects model are presented in Table 3. Model 1 showed that when participants were looking at pictures of seven items (vs. two), a sentence with two plural morphemes (vs. a sentence with one plural morpheme) led to significantly more “yes” decisions (high vs. low numerosity–high vs. low morphology). Participants also made significantly more “yes” decisions for sentences with one plural morpheme (vs. none) combined with pictures of seven items (vs. two) (high vs. low numerosity–low vs. no morphology). In sum, morphological and conceptual information interacted in participants' decisions that the sentence was an acceptable match for the picture. Figure 2 shows the percentage of “yes” decisions by conceptual and morphological condition.

**Table 3:** Results of model 1: acceptability decision.

**Variable**	**β**	**SE**	***t***	***p***
Intercept	0.675	0.035	19.417	<0.001
0 vs. 1 plural morpheme	0.083	0.089	0.935	n.s.
1 vs. 2 plural morphemes	0.043	0.089	0.486	n.s.
1 vs. 2 items pictured	0.421	0.042	10.005	<0.001
2 vs. 7 items pictured	0.268	0.042	6.367	<0.001
0 vs. 1 plural morpheme:1 vs. 2 items pictured	1.461	0.125	11.731	<0.001
0 vs. 1 plural morpheme:2 vs. 7 items pictured	0.816	0.125	6.555	<0.001
1 vs. 2 plural morphemes:1 vs. 2 items pictured	0.982	0.125	7.885	<0.001
1 vs. 2 plural morphemes:2 vs. 7 items pictured	0.77	0.125	6.180	<0.001

### 5.2. Accuracy

**Figure 3:**
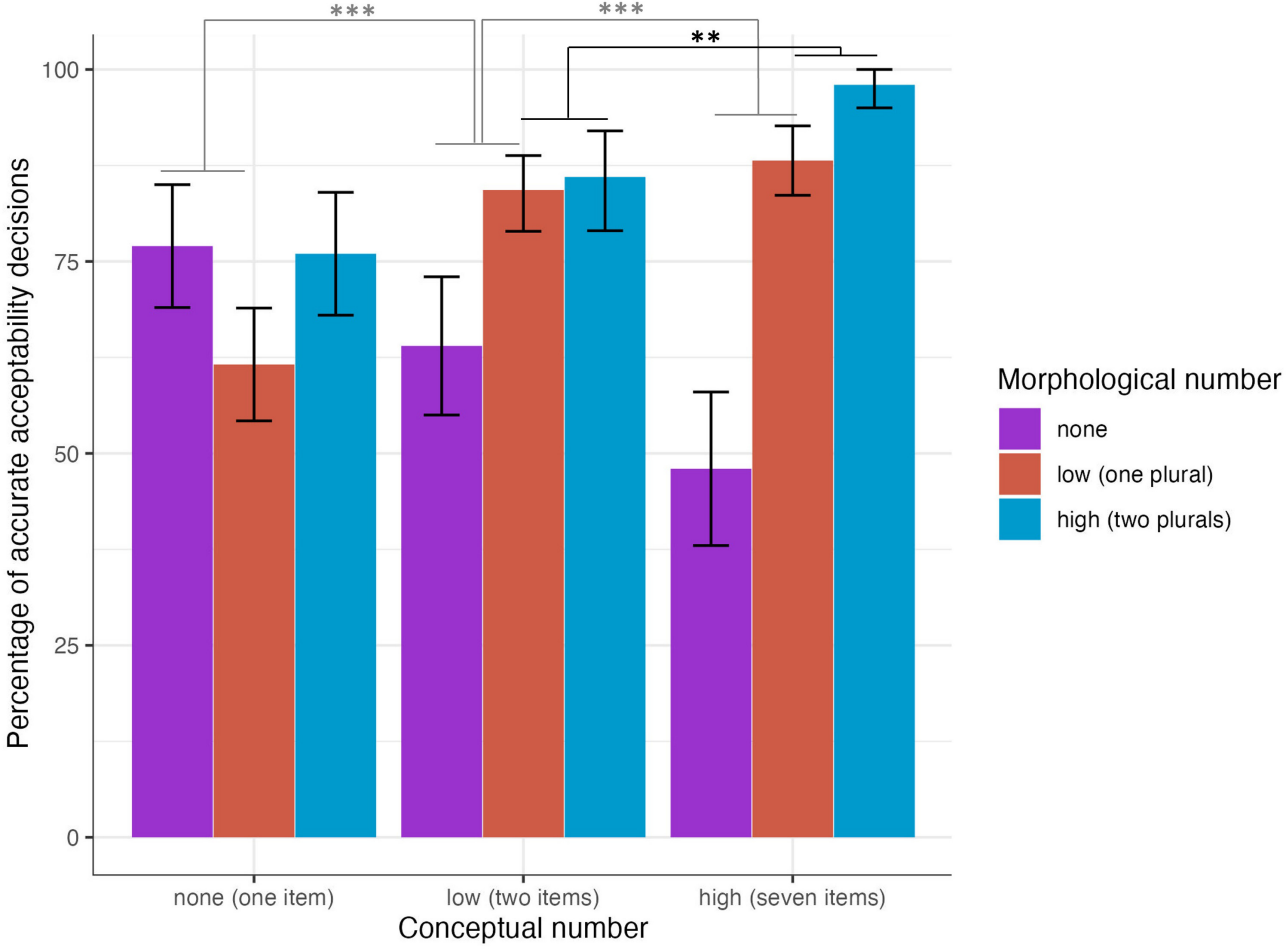
Percentage of accurate decisions by morphological condition for the two and seven conceptual conditions (error bars indicate bootstrapped 95% confidence intervals). *p < 0.05, **p < 0.01, ***p < 0.001.

Model 2 (shown in Table 4 below) investigated to what extent morphological and conceptual information predicted a participant's accuracy (“correct/accurate” vs. “incorrect/inaccurate”) that the auditorily presented sentence matched the picture shown. Based on prior work examining optional plural marking with native speaker judgements (Butler, 2012) and sentence production experiments (Butler and Couoh Pool, 2018), the only strictly inaccurate responses would be: (1) a yes response to a picture of one item with any plural morphology or (2) a no response to a picture of two or seven items. All other responses are assumed to be accurate. We analyze accuracy based on these assumptions, but we also consider the possibility that the acceptability of sentences is likely to vary across participants especially in a population less familiar with psycholinguistic experiments and testing paradigms in general (Henrich et al., 2010). When participants were looking at pictures of seven items (vs. two), a sentence with two plural morphemes (vs. a sentence with one plural morpheme) led to significantly more accurate decisions. Participants made significantly more accurate decisions for sentences with one plural morpheme (vs. none) combined with pictures of seven items (vs. two) (high vs. low numerosity–low vs. no morphology). The percentage of accurate decisions is shown in Figure 3 by morphological and conceptual conditions.

**Table 4:** Results of model 2: accuracy.

**Variable**	**β**	**SE**	***t***	***p***
Intercept	0.758	0.030	24.877	<0.001
0 vs. 1 plural morpheme	0.25	0.090	2.773	<0.01
1 vs. 2 plural morphemes	0.221	0.090	2.454	<0.05
1 vs. 2 items pictured	0.120	0.045	2.697	<0.01
2 vs. 7 items pictured	0.116	0.045	2.596	<0.01
0 vs. 1 plural morpheme:1 vs. 2 items pictured	0.858	0.132	6.506	<0.001
0 vs. 1 plural morpheme:2 vs. 7 items pictured	0.521	0.132	3.963	<0.001
1 vs. 2 plural morphemes:1 vs. 2 items pictured	0.13	0.132	0.985	n.s.
1 vs. 2 plural morphemes:2 vs. 7 items pictured	0.358	0.132	2.717	<0.01

### 5.3. Decision time

Each participant's decision time was subtracted from the length of the auditorily presented stimulus sentence to standardize over sentences having slight variations in length due to the absence or presence of plural morphology. For the analysis of decision time, we analyzed only “yes” decisions because we are most interested in the decision times for pictures of more than one item. To clarify further, since plural marking is optional, the only conditions in which we expect a decision of “no” are those showing a picture of one nominal referent paired with a sentence containing any plural marking. In addition, including acceptability decision in the model as a covariate resulted in over-parameterization. Table 5 shows the mean and standard deviation for the length in seconds of the stimulus sentences in each condition, the total decision time and the adjusted decision time (calculated as the total decision time subtracted from the stimulus sentence time). Overall, the two plural condition resulted in faster decisions compared to the one plural and no plural conditions.

**Table 5:** Stimulus sentence time, total decision time, and adjusted decision time in seconds.

**Condition**	**Stimulus sentence time**	**Total decision time**	**Adjusted decision time**
	**M (SD) seconds**	**M (SD) seconds**	**M (SD) seconds**
No plural	3.03 (0.36)	5.56 (3.03)	2.54 (3.00)
One plural	3.33 (0.27)	5.75 (4.72)	2.42 (4.71)
Two plurals	3.32 (0.26)	5.03 (2.08)	1.71 (2.18)

**Figure 4:**
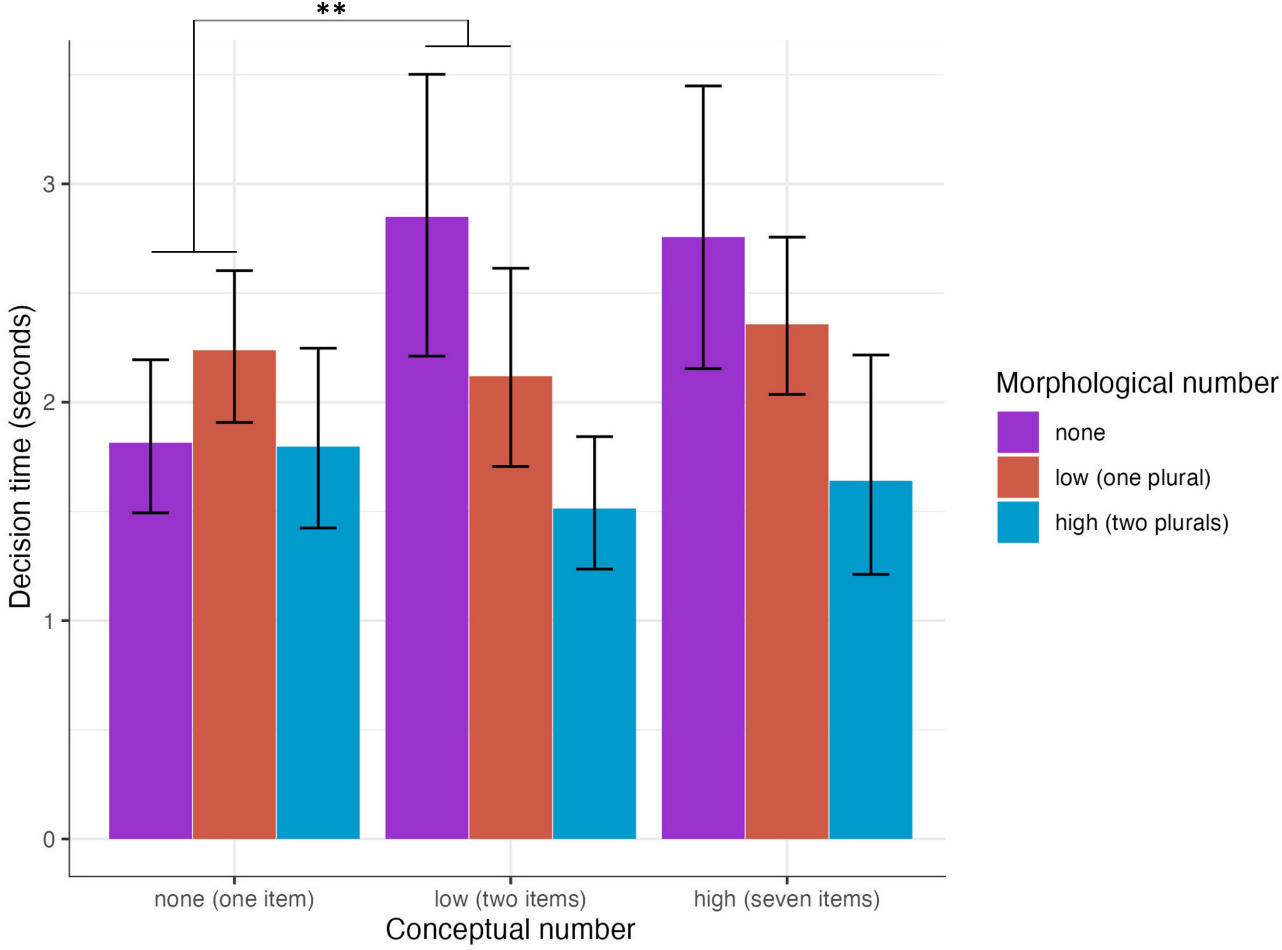
Decision time for “yes” decisions (seconds after the end of the sentence) for accurate decisions by morphological condition for the two and seven conceptual conditions (error bars indicate bootstrapped 95% confidence interval).

Model 3 (shown in Table 6) reports the results of the mixed effects linear regression. Figure 4 shows the mean decision times by conceptual and morphological conditions. In Model 3, morphology but not numerosity predicted decision time. There was a significant interaction between no plural and one plural morpheme with one vs. two items pictured. No plural marking led to significantly faster decisions in the one item condition compared to the two item condition, but for one plural morpheme, there was no difference between the one and two item conditions. No other effects were significant.

**Table 6:** Results of model 3: decision time.

**Variable**	**β**	**SE**	***t***	***p***
Intercept	2.11	0.21	10.122	<0.001
0 vs. 1 plural morpheme	−0.73	0.29	−2.497	<0.05
1 vs. 2 plural morphemes	−0.94	0.29	−3.208	<0.01
1 vs. 2 items pictured	0.37	0.24	1.565	n.s.
2 vs. 7 items pictured	0.24	0.24	1.000	n.s.
0 vs. 1 plural morpheme:1 vs. 2 items pictured	−1.98	0.71	−2.787	<0.01
0 vs. 1 plural morpheme:2 vs. 7 items pictured	−0.67	0.71	−0.943	n.s.
1 vs. 2 plural morphemes:1 vs. 2 items pictured	−1.08	0.71	−1.521	n.s.
1 vs. 2 plural morphemes:2 vs. 7 items pictured	−0.34	71	−0.485	n.s.

## 6. Discussion

In this sentence comprehension experiment with speakers of Yucatec Maya, numerosity and morphology interacted to predict the likelihood of a participant's decision that a picture and a sentence were an acceptable match. When participants were looking at pictures of seven items (vs. two), a sentence with two plural morphemes (vs. a sentence with one plural morpheme) led to significantly more “yes” decisions that the sentence and the picture were an acceptable match. Participants also made significantly more “yes” decisions for sentences with one plural morpheme (vs. none) combined with pictures of seven items (vs. two). The results of the accuracy analysis showed a similar pattern of interactions. When participants were looking at pictures of seven items (vs. two), a sentence with two plural morphemes (vs. a sentence with one plural morpheme) led to significantly more accurate decisions. Similarly, participants made significantly more accurate decisions for sentences with one plural morpheme (vs. none) combined with pictures of seven items (vs. two). The results of the decision time data, however, diverge from the interactions of morphological and conceptual information observed in the decision and accuracy data. Participants made significantly faster “yes” decisions when a sentence had two plural morphemes vs. one and when a sentence had one plural morpheme compared to none.

These effects of conceptual information in sentence processing in Yucatec Maya can also be seen in the domain of sentence production. In a previous psycholinguistic study on the role of numerosity in sentence production with speakers of Yucatec Maya, an increase in numerosity (pictures of one, two and seven humans or animals) significantly increased the likelihood of optional plural morphology use on the noun (Butler and Couoh Pool, 2018). In the current study on the role of numerosity in sentence comprehension with speakers of Yucatec Maya, conceptual and morphological number interacted such that high conceptual information in combination with high morphological number information affected a participant's decision that a sentence was an acceptable match for a picture. On the other hand, morphological information alone predicted significantly faster acceptability decisions.

As outlined in the introduction, cross-linguistic research investigating the morphological richness hypothesis has resulted in two different findings: (1) languages with richer morphological paradigms will be more susceptible to the conceptual influences of number due to the salient role of meaning in the computation of number morphology or (2) languages with richer morphological paradigms will be less susceptible to the conceptual influences of number due to the salience of grammatical specifications of plural morphemes which cancel the effects of number meaning. The goal of this study was to examine this question in Yucatec Maya, a language with optional plural morphology. Previously reported data on the effects of conceptual number on the production of plural morphology in Yucatec Maya showed that despite Yucatec Maya being a language that lacks a rich morphological paradigm for number, participants were significantly more likely to produce the optional plural when describing pictures of two items vs. one and when describing pictures of seven items vs. two (Butler and Couoh Pool, 2018). In the replication of the sentence production findings in the domain of sentence comprehension, the present study found conceptual factors to interact with morphological factors in facilitating sentence comprehension in decisions and accuracy. Taken together, this suggests that conceptual number information plays a significant role in sentence production and comprehension in Yucatec Maya even in light of low morphological richness for number. This result from Yucatec Maya supports the second approach to morphological richness–a language with less rich number morphology will show increased effects of conceptual number as there is no inflectional number paradigm by which number meaning is grammatically expressed (Foote, 2006; Lorimor et al., 2008).

The decision time findings, however, raise questions about the second approach. In terms of the decision time data, a measure that is perhaps more sensitive to real-time incremental sentence comprehension, the presence of the optional plural morpheme led to faster decisions. Since plural morphology is optional, this finding may go against an approach to sentence comprehension arguing that languages with less rich morphological paradigms show increased effects of conceptual number across the board. However, this result aligns with some findings from the broader psycholinguistic literature on morphological and syntactic processing. Psycholinguists have long been interested in what information is available at different processing stages whether the approach taken is modular, with distinct levels of conceptual, functional and positional processing (Garrett, 1976, 2000; Levelt, 1989) or one that presumes information can spread more freely between levels (Vigliocco and Franck, 2001) or as cascading activation and connectionist models (Dell, 1986; Caramazza, 1997; Dell et al., 1999; Chang et al., 2006). Agreement processing has provided a window into what information is available during positional processing, the stage at which inflectional morphology is hypothesized to take place. The results from Yucatec Maya suggest that cross-linguistic differences are possible in the flow of information during sentence production and comprehension, a conclusion that is supported by research on agreement in noun phrase domain (Costa, 1999; Schiller and Caramazza, 2003, *inter alia*).

We found that decision times were the fastest when plural morphemes were present, and the sentences with a plural marked noun and a plural marked verb led to the fastest decisions. While this finding was not central to the hypotheses being tested, it is somewhat surprising. If plural marking is optional, why would adding this optional morphological complexity facilitate comprehension. In the broader context of theories of sentence comprehension, this finding aligns with the identification of the controller of agreement at the locus of the verb (Badecker and Kuminiak, 2007; Wagers et al., 2009). In Yucatec Maya, when an optional plural is present on the verb, it triggers a search through working memory to retrieve the subject, or controller of agreement, which is more easily retrieved when it is also plural-marked. While this study was not designed to test this theory, the result that decision times were faster with two plurals aligns with this account. In contrast, it would not align well with the theories, e.g., Marking and Morphing (Eberhard et al., 2005) which posit that number information on the head noun is faulty or ambiguous (Franck et al., 2002; Eberhard et al., 2005; Staub, 2009), as we found differences between conditions in which nouns were unmarked as well as marked.

Regardless of approach, theories that better integrate features of morphological processing (Amenta and Crepaldi, 2012) in sentence processing paradigms are necessary to advance psycholinguistic research on typologically diverse languages, as morphology tends to be highly language-specific but mechanisms that drive morphological processing may be similar to those that drive sentence processing (Leminen et al., 2019).

Two language-specific explanations are relevant to this discussion as well. The first potential alternative explanation is that Yucatec Maya is a language that is in the process of changing from a canonical verb-subject-object (VSO) language, like several other Mayan languages, to a subject-verb-object (SVO) language, which is associated with inflectional subject-verb agreement paradigms, one in which verbal marking covaries with marking on the subject (Gutiérrez-Bravo and Madera, 2010; England, 2011). This would align with the finding that sentences with plural marking on the noun and verb led to faster decisions.

The second potential alternative explanation is that plural morphology on the noun plays a different role than plural morphology on the verb. Nominal plural indicates a non-singleton nominal referent. In subject-agreement languages, plural morphology co-varies on the verb with nominal plural marking. In several Mayan languages, however, plural morphology in the verbal domain marks pluractionality, that the action expressed by the verb is repeated independent of the numerosity of the nominal referent (Henderson, 2019). For example, the sentence “the girls are fishing” could indicate that all girls have their own fishing poles and are casting. On the other hand, a non-pluractional interpretation would be that the girls as a group are fishing, but not every girl is holding a fishing pole and casting (some may be watching while part of the group). All of the stimuli presented in this study depicted pluractionality, so future studies would be necessary to answer this question. More generally, in the pursuit of cross-linguistic psycholinguistics with typologically diverse, less commonly studied languages, careful understanding of the linguistic features of the language, including collaborations between linguists and psycholinguists, will be fundamental. Future studies should address these and related questions. To truly encompass cross-linguistic variation in psycholinguistics, new questions that a-priori appear to have little to no relevance to the languages commonly studies by psycholinguists must be explored.

Overall, this study expands the empirical base of linguistic and psycholinguistic studies of typologically diverse languages to improve the understanding of an under-studied language and explore the implications for universal approaches to modeling human sentence production and comprehension. The phenomena of number marking and agreement processes are generally understudied in the field of Mayan linguistics (England, 2011). Moreover, the need to expand the focus to non-Indo-European languages has been continuously highlighted in the field of psycholinguistics (Christianson and Ferreira, 2005; Norcliffe, 2009; Norcliffe et al., 2015b) and across the larger domain of cognitive sciences (Henrich et al., 2010).

## Data availability statement

The data are available from the author upon request. The experimental materials are available at https://github.com/lkbutler3/yucatec-comp/.

## Ethics statement

The studies involving humans were approved by Pennsylvania State University Institutional Review Board. The studies were conducted in accordance with the local legislation and institutional requirements. The ethics committee/institutional review board waived the requirement of written informed consent for participation from the participants or the participants' legal guardians/next of kin because the study was determined to be low risk and it was conducted in Yucatec Maya language, a language which few people read and write. Many speakers of Yucatec Maya do not read or write in Spanish either. Participants gave verbal assent in Yucatec Maya with a trained Yucatec Maya-speaking consultant who was a member of the community. For minors, verbal assent was obtained in Yucatec Maya from the minor and their parent or caregiver.

## Author contributions

LB contributed to all aspects of study design, data collection, analysis, writing, editing, and funding acquisition.

## Footnotes

^1^Abbreviations used include: 3 - third person, A - Set A cross-reference marker, DEF - definite article, D2 - distal deictic marker, INC - incompletive status marker, PL - plural, PROG - progressive aspect, SG - singular.
